# A molecular beacon real-time polymerase chain reaction assay for the identification of *M. chitwoodi*, *M. fallax*, and *M. minor*

**DOI:** 10.3389/fpls.2023.1096239

**Published:** 2023-02-22

**Authors:** Scott D. Anderson, Cynthia A. Gleason

**Affiliations:** Department of Plant Pathology, Washington State University, Pullman, WA, United States

**Keywords:** meloidogyne, nematode, diagnostic tool, RT-PCR, potato, pathogen detection

## Abstract

Root-knot nematodes (*Meloidogyne* spp.) are major pests of many important crops around the world. In the Northwestern region of the United States of America (USA), *Meloidogyne chitwoodi* causes economic losses in potatoes because the nematodes can infect the tubers, which leads to potato galling and reductions in marketable yield. *Meloidogyne chitwoodi* is a quarantine pathogen in certain potato export markets, and there is little industry tolerance for the presence of this nematode. Recently, two *Meloidogyne* species that are not known to be present in agricultural fields in the USA were detected on golf turfgrasses in California and Washington. These species, *M. fallax* and *M. minor*, are morphologically similar to *M. chitwoodi* and can infect potatoes and cause tuber damage. Their detection in the USA means that they could potentially infest potato fields and become a problem in potato production. Additionally, *M. fallax* is a regulated plant pest in the USA, which makes the correct identification of potato-infecting root-knot nematodes important. Previously, there was no single-tube assay that could determine whether *M. chitwoodi*, *M. fallax*, and/or *M. minor* were present in a sample. Thus, a molecular beacon real-time PCR assay which can reliably detect *M. chitwoodi*, *M. fallax*, or *M. minor* from crude nematode extracts was designed and characterized.

## Introduction

Root-knot nematodes are obligate sedentary endoparasites of many important agricultural crops, causing upwards of $180 billion in global crop losses annually (Sasser and Freckman, 1987; Koenning et al., 1999). Root-knot nematodes are especially problematic in potato because the nematodes cause tuber galling and internal tuber defects (Lima et al., 2018; Bali et al., 2021a). In 2019, the United States had the 5th highest production of potatoes (FAO, 2017), with three states, Oregon, Washington, and Idaho, comprising over half of the country’s entire production (Zasada et al., 2018). These states have the root-knot nematodes *M. chitwoodi* and *M. hapla*. However, *M. chitwoodi* is a larger threat in potato production because it hatches at lower temperatures, which allows its populations to expand rapidly in a single growing season. *Meloidogyne chitwoodi* also causes more visible galling on potatoes compared to *M. hapla* (Pinkerton et al., 1991; Ingham et al., 2000). The galling causes the tuber surface to look bumpy, and necrotic dark spots form in the tuber flesh around the *M. chitwoodi* females. These visual defects can significantly decrease the potato market value due to the near zero tolerance to tuber blemishes in the processing market. Although *M. chitwoodi* is endemic to the western region of the United States, its regulated status means there is a zero- tolerance policy in potatoes destined for export to several world markets (Ingham et al., 2007). There is currently no genetic resistance against root-knot nematodes in commercial potato cultivars.

In Europe, root-knot nematodes that are commonly found in cooler climates include *M. hapla, M. naasi, M. chitwoodi* and *M. fallax* (Wesemael et al., 2011), and both *M. chitwoodi* and *M. fallax* are root-knot nematode species on the A2 EPPO alert list (Wesemael et al., 2011; Viaene, 2014; European and Mediterranean Plant Protection Organization [EPPO], 2022). *Meloidogyne fallax* was originally identified as *M. chitwoodi*, but subsequent morphological and biochemical investigations revealed that it was a distinct species (Karssen, 1996). *Meloidogyne fallax* and *M. chitwoodi* are difficult to distinguish morphologically. They also have some of the same hosts, such as potatoes and carrots, but they differ in their ability to infect some crops, such as corn (Karssen, 1996). Both *M. chitwoodi* and *M. fallax* can cause significant damage to potato tubers, and some data indicate that *M. fallax* is more aggressive on potato than *M. chitwoodi* (Van Meggelen et al., 1994; Van Der Beek et al., 1998; Suffert and Giltrap, 2012). Although *M. fallax* has not been reported on potato in the USA, it was detected during a survey of golf course greens in California (Nischwitz et al., 2013). Follow up surveys by APHIS in California did not detect *M. fallax*, so it is considered “not present” in the USA (Kantor et al., 2022), but there remains a threat of introduction.

In 2000 a new species of root-knot nematode called *M. minor* was found on heavily infected potato plants from a potato field in Zeijerveld, The Netherlands (Karssen et al., 2004). The potato tubers exhibited pimple-like galling similar to the symptoms caused by *M. chitwoodi* and *M. fallax*. *Meloidogyne minor* was subsequently found on golf courses in the Netherlands, Belgium, United Kingdom, and Ireland (Vandenbossche et al., 2011; Morris et al., 2013). Interestingly, *M. minor* infections did not significantly impact tuber yield or quality in two potato cultivars (cvs Astérix and Markies) in field plots in the Netherlands (Thoden et al., 2012). However, significant tuber damage was inflicted by *M. minor* on potatoes grown in greenhouses (Thoden et al., 2012), indicating that *M. minor* biology and pathogenicity at relatively warmer temperatures, such as those in greenhouses, are not fully understood. There is also limited information about *M. minor* infectivity on potato cultivars commonly used in the USA, such as the Russets. Recently *M. minor* was reported on turf grass in the USA (McClure et al., 2012; Nischwitz et al., 2013). It is possible that this nematode could be accidentally transmitted from golf courses to arable land *via* contaminated sports shoes or equipment (Morris et al., 2011; Morris et al., 2013). With the potential for *M. minor* to spread and cause damage to potatoes, it is prudent to monitor for *M. minor* to help mitigate the risk it poses to USA agriculture.

To ensure proper monitoring of these *Meloidogyne* species, a rapid and reliable molecular test to identify these three species is necessary. Many molecular techniques have been established for identifying *Meloidogyne* species (Baum et al., 1994; Vrain and Petersen, 1996; Blok et al., 1997; Petersen et al., 1997; Williamson et al., 1997; Zijlstra, 1997; Castagnone-Sereno et al., 1999; Castagnone-Sereno, 2000; Zeng et al., 2015; Zhang and Gleason, 2019). These molecular techniques were often based on PCR using species specific primers that target the mitochondrial DNA, or they target the intergenic region or the internal transcribed spacer (ITS) regions of the ribosomal DNA (Giorgi et al., 1994; Hadziavdic et al., 2014; Harris et al., 1990; Van Megen et al., 2009). The technique called PCR-RFLP, which is PCR followed by restriction digestion of the amplicons to create unique restriction fragment lengths, can be used to identify several *Meloidogyne* species (Harris et al., 1990; Vrain et al., 1992; Powers and Harris, 1993; Zijlstra et al., 1995; Powers et al., 1997; Orui, 1998; Waeyenberge et al., 2000; Han et al., 2004; Gamel et al., 2014). The technique was found to be sensitive enough to detect a single juvenile of *M. fallax* or *M. chitwoodi*, but PCR-RFLP results can be difficult to interpret when there are mixtures of species in the reaction (Gamel et al., 2014). Zijlstra (2000) showed that primers designed to amplify sequence-characterized amplified regions (SCARs) could be used in SCAR-PCR to identify *M. hapla*, *M. chitwoodi* and *M. fallax*. The drawback to the SCAR-PCR is that it is not as sensitive as PCR-RFLP, and it requires at least two juveniles of *M. chitwoodi* or one juvenile of *M. fallax* as template (Zijlstra, 2000). Moreover, the previously designed SCAR-PCR primers for *M. fallax* were shown to cross-react with *M. minor* allowing for false-positives (Nischwitz et al., 2013). A multiplex real-time PCR (TaqMan) using primers designed for the ITS region was developed for the simultaneous detection of *M. chitwoodi* and *M. fallax* (Zijlstra and Van Hoof, 2006). The TaqMan PCR was a breakthrough in root-knot nematode identification in multiplexed reactions, but it did not include a third nematode of interest, *M. minor* (Zijlstra and Van Hoof, 2006; Wesemael et al., 2014).

There are no molecular techniques that can identify and distinguish *M. chitwoodi*, *M. fallax*, and *M. minor* in a one tube assay. With this in mind, we have developed a new RT-PCR assay using molecular beacons to identify and distinguish *M. chitwoodi*, *M. fallax*, and *M. minor* in a single reaction. This assay can be used by regulatory agencies and diagnosticians to monitor for the presence of these potato-infecting nematodes, two of which are of regulatory importance, using as little as a single juvenile isolated from samples.

## Materials and methods

### *In silico* design of primers and beacons

Primers and beacons were designed using Beacon Designer 8 (Premier Biosoft, Palo Alto, CA). Alignments of *HSP90* gene sequences (**Table 1**) were used to find universally conserved primer sequences surrounding polymorphic regions in *M. chitwoodi*, *M. fallax*, and *M. minor* (Skantar and Carta, 2004). To ensure the beacon probes and primers were specific to their target organisms, the sequences were queried using the BLASTn search program and the non-redundant database (National Center for Biotechnology Information, NCBI). Primers and the beacons for the *HSP90* region were synthesized by Sigma-Aldrich (St. Louis, Mo) and are described in **Table 2**. The *M. chitwoodi* beacon has 6-FAM, *M. fallax* beacon has HEX, and *M. minor* beacon has Cyan-5 as the reporters. For the quenchers, the Black Hole Quencher (BHQ-1 for 6-FAM and HEX, and BHQ- 3 for Cyan-5) was used.

**Table 1:** *Meloidogyne* spp. *HSP90* regions used for beacon designs.

Species	Genebank ID
*M. chitwoodi*	KC262220.1
	KC262221.1
	KC262222.1
	KC262223.1
	KC262224.1
*M. fallax*	KC262225.1
	KC262226.1
	KC262227.1
	KC262228.1
	KC262229.1
	KC262233.1
*M. minor*	KC262234.1
	KC262235.1
	KC262236.1
	KC262237.1
	KC262238.1
	KC262239.1
	KC262240.1
	KC262241.1
	KC262242.1
	KC262243.1
	KC262244.1
	KC262245.1
	KC262246.1

**Table 2:** Primers and beacon probes used in the molecular beacon RT-PCR assays.

Name	Fluorophore	Sequence
Mc-complementary	–	AGGATGCAAGATTTAAGGCAAT
Mf-complementary	–	GTTGTGATAAGTAGAAGGCAAGA
Mm-complementary	–	AATTGACCCTCAACGCTC
Mc-SNP	–	TAGGATGCAAG**T**TTTAAGGCAAT
Mf-SNP	–	GTTGTGATAAGT**T**GAAGGCAAGA
Mm-SNP	–	AATTGACC**T**TCAACGCTC
F-HSP90	–	AGCTTGTCTAATGATTGG
R-HSP90	–	GGAACAAACAAAAGAGCT
C1-HSP90-FAM-6	FAM	GCGATCTAGGATGCAAGATTTAAGGCAATGATCGO
F1-HSP90-HEX-5	HEX	CGATCGTTGTGATAAGTAGAAGGCAAGAGATCG
M2-HSP90-Cyan-5	Cyan5	CGATAATTGACCCTCAACGCTCATCG

### Thermal denaturation curves

Three synthetic oligos complementary to each beacon’s loop sequence and three off-target synthetic oligos containing a single nucleotide difference to the loop sequence were synthesized by Sigma-Aldrich (**Table 2**) and used for creating thermal denaturation curves. For each beacon, two 50 µL reactions containing 200 nM molecular beacon probe in 3 mM MgCl2 and 10 mM Tris- HCl, pH = 8.0 were prepared with 600 nM of either the complementary or off-target synthetic oligo. A third reaction for each beacon was run containing Milli-Q water in place of the synthetic oligo as a control. Reactions were prepared in a 96-well clear qPCR plate and run on a CFX96 Real-Time PCR Detection System (Bio-Rad Laboratories, Hercules, CA) starting at 80°C and decreasing to 30°C at a rate of 1°C/minute, with fluorescence measured every minute.

### Nematode templates for PCR

For genomic DNA templates, the DNA was extracted from *M. chitwoodi* (Race 1, Race 2, and Roza) (Bali et al., 2021b; Mojtahedi et al., 2007), *M. incognita, M. javanica, M. arenaria*, and *M. hapla* eggs using a phenol/chloroform extraction protocol as described by Gross and Williamson (2011). All three *M. chitwoodi* isolates (Race 1, Race 2, and Race 1 Roza) were originally provided by Dr. Charles Brown (USDA-ARS). The *M. hapla* isolate VW9, *M. incognita* isolate VW6, *M. javanica* isolate VW4, and *M. arenaria* isolate HarA were provided by Dr. Valerie Williamson (**Table 3**, UC-Davis). All *Meloidogyne* species were maintained on the susceptible tomato *Solanum lycopersicum* cv. Rutgers under greenhouse conditions. The identities of the *M. chitwoodi, M. hapla*, *M. incognita*, *M. javanica* and *M. arenaria* populations were confirmed using species-specific PCR (Powers and Harris, 1993; Zijlstra, 2000; Wishart et al., 2002).

**Table 3:** *Meloidogyne* spp. used for testing molecular beacon RT-PCR specificity.

Species	Race/Strain	Source
*M. chitwoodi*	Race 1	WA, USA
*M. chitwoodi*	Race 2	WA, USA
*M. chitwoodi*	Roza	WA, USA
*M. fallax*	–	Netherlands
*M. minor*	–	Netherlands
*M. hapla*	VW9	CA, USA
*M. incognita*	VW6	CA, USA
*M. javanica*	VW4	CA, USA
*M. arenaria*	EC2	CA, USA

To amplify and clone the *HSP90* amplicon from *M. chitwoodi* race 1, *M. fallax* and *M. minor*, F- HSP90 and R-HSP90 primers were used in a PCR with approximately 15 ng of *M. chitwoodi, M. fallax*, or *M. minor* DNA. Reactions were 50 µL total volume and contained 1x AmpliTaq Gold 360 buffer, 3 mM MgCl2, 200 µM dNTPs, 200 nM F-HSP90 and R-HSP90 primers, and 1.25 U of AmpliTaq Gold. The reactions were run on an Eppendorf Mastercycler Pro Thermal Cycler as follows: 94°C for 10 minutes, then 30 cycles of 94°C for 15 seconds, then 58°C for 30 seconds, and 72°C for 30 seconds, and then finally 72°C for 5 minutes. Reactions were pooled and cleaned up using a ThermoFisher GeneJET PCR purification kit (Thermo Fisher, Waltham, MA) and the purified PCR products were ligated into pGEM-T vector. The 10 µL ligation reaction contained 1x Rapid Ligation buffer, 3 Weiss Units of T4 ligase, purified PCR product, pGEM-T vector (Promega, Madison, WI), and Milli-Q water. Plasmids were cloned into TOP10 *E. coli* and extracted from the overnight bacterial cultures using a GenElute Plasmid Miniprep kit (Sigma-Aldrich, St. Louis, MO). The *HSP90* amplicons from *M. chitwoodi, M. minor*, and *M. fallax* in the pGEM-T vector will be referred to as plasmid *HSP90.*

### Optimizing the reactions

A standard curve using *M. chitwoodi* gDNA as template was prepared using 1:10 serial dilutions. Reactions were run at a volume of 50 µL on a CFX96 Real-Time PCR Detection System in a 96-well clear qPCR plate. Reaction mixes contained 1x AmpliTaq Gold 360 buffer, 3 mM MgCl2, 200 µM dNTPs, 200 nM F-HSP90 and R-HSP90 primers, 200 nM target beacon (C1-Hsp90-FAM-6) and 1.25 U of AmpliTaq Gold. The cycling conditions were as follows: 95°C for 10 minutes, then 55 cycles of 95°C for 15 seconds (sec), 54°C for 30 sec, and 72°C for 15 sec. The increase in fluorescent signal was registered during the annealing step of the reaction. The *M. chitwoodi* standard curve used 5 points, with template DNA concentrations ranging from 40 ng to 4 pg. Additional standard curves were made for *M. chitwoodi, M. fallax* (F1-Hsp90-HEX-5 beacon), and *M. minor* (M2-Hsp90-Cyan-5 beacon) using the same reaction conditions, but in which the plasmid *HSP90* templates for the reactions were serial dilutions of 0.4 ng – 4 fg, 2 ng – 2 fg, and 0.4 ng – 0.04 fg respectively. Each concentration was run in triplicate. Non-template controls containing only water were included for all standard curves, and each curve was repeated with similar results. To calculate the PCR efficiency, the following equation was used


$$\text{PCR efficiency }\%\;=\;(10\frac{1}{\bar{s}lope}\;-\;1)*100$$

### Molecular beacon RT-PCR

The second stage juveniles (J2s) of *M. chitwoodi* race 1 were hatched from eggs collected from Rutgers tomatoes grown in greenhouses at Washington State University. *Meloidogyne fallax* and *M. minor* J2s were provided by the Wageningen Nematode Collection (National Plant Protection Organization, the Netherlands) and stored in DESS at -20°C. Second stage juveniles were digested using the protocol described in Qiu et al. (2006). In brief, J2s were picked under a dissecting scope and transferred to 15 µL droplets of Milli-Q water. The nematodes were crushed using a pipet tip; 10 µL of the crushed nematode was transferred to a 10 µL solution containing 2 µL AmpliTaq Gold 360 buffer, 2 uL of 600 µg/mL Proteinase K, and 6 µL Milli-Q water. The reaction mixes were incubated at -20°C for 20 minutes, heated for 1 hour at 65°C, and then 95°C for 10 min before cooling to room temperature. The nematode samples were spun at 12,000 rpm for 2 min before storing at -20°C until used in PCR. Five µL of these J2 samples were used as template for molecular beacon RT-PCR, and each sample was run in duplicate or triplicate. The 50 µL reaction mixes contained 1x AmpliTaq Gold 360 buffer, 3 mM MgCl2, 200 µM dNTPs, 200 nM F-HSP90 and R-HSP90 primers, 200 nM target beacon (C1-Hsp90-FAM-6, F1-Hsp90-HEX-5, or M2-Hsp90-Cyan-5) and 1.25 U of AmpliTaq Gold. The reaction conditions were the same as those used for the standard curves. All molecular beacon RT-PCR assays with these three species were repeated at least twice with similar results.

To ensure the specificity of this assay 40 ng of gDNA from *M. incognita*, *M. javanica*, *M. arenaria*, or *M. hapla* was used as template for molecular beacon RT-PCR assay using C1-Hsp90-FAM-6, F1-Hsp90-HEX-5, and M2-Hsp90-Cyan-5 beacons. Non-template controls containing only Milli- Q water were included as well as positive controls using 4 pg of plasmid *HSP90* for *M. chitwoodi*,

*M. fallax*, or *M. minor*. The 50 µL reaction mixes contained 1x AmpliTaq Gold 360 buffer, 3 mM MgCl2, 200 µM dNTPs, 200 nM F-HSP90 and R-HSP90 primers, 200 nM target beacon (C1- Hsp90-FAM-6, F1-Hsp90-HEX-5, or M2-Hsp90-Cyan-5) and 1.25 U of AmpliTaq Gold. The reaction conditions were the same as those used for the standard curves. The molecular beacon RT-PCR assays with non-target species were repeated twice with similar results.

All PCR products in this paper were visualized as follows: 10 µL of each PCR product was loaded onto a 1.5% agarose gel and separated for 45 min at 100 V before visualizing with ethidium bromide under UV light. The Invitrogen 1-kb plus DNA ladder was used as reference for size.

### Multiplex molecular beacon RT-PCR

Initial multiplex molecular beacon RT-PCR assays were performed using equal template concentrations of each species. Each reaction tube contained the same amount of *M. chitwoodi, M. minor* and *M. fallax* template *HSP90* plasmid DNA (20 pg of each species). To the template DNAs, the reaction mix was added. The reaction mix contained 1x AmpliTaq Gold 360 buffer, 3 mM MgCl2, 200 µM dNTPs, 200 nM F-HSP90 and R-HSP90 primers, 200 nM of each of the three target beacons (C1-Hsp90-FAM-6, F1-Hsp90-HEX-5, and M2-Hsp90-Cyan-5) and 1.25 U of AmpliTaq Gold polymerase. The amount of fluorescence for each reaction at different template concentrations with the three different probes was measured at each cycle on the CFX96 Real- Time PCR Detection System.

Varying ratios of plasmid *HSP90* templates were used to test the validity of multiplexing. One template was kept at 20 pg while the other two were loaded ten times more concentrated at 200 pg each. Reaction mixes contained 1x AmpliTaq Gold 360 buffer, 3 mM MgCl2, 200 µM dNTPs, 200 nM F-HSP90 and R-HSP90 primers, 200 nM of each beacon (C1-Hsp90-FAM-6, F1-Hsp90-HEX-5, M2-Hsp90-Cyan-5) and 1.25 U of AmpliTaq Gold. The reaction conditions were the same as those used for the standard curves.

Multiplex with proteinase K digested J2s were tested with 1 J2 or 5 J2 of each species present in the reaction mix. A single J2 from *M. chitwoodi*, *M. fallax*, and *M. minor* were picked under a dissecting microscope and placed into a single 15 µL droplet of Milli-Q water and digested as described above and used as template for qPCR. Similarly, for the 5 J2 multiplexing, 5 J2s from *M. chitwoodi*, *M. fallax*, and *M. minor* were picked under a dissecting microscope and placed into a single 15 µL droplet of Milli-Q water and digested and used as template for qPCR. Reaction mixes contained 1x AmpliTaq Gold 360 buffer, 3 mM MgCl2, 200 µM dNTPs, 200 nM F-HSP90 and R-HSP90 primers, 200 nM of each beacon (C1-Hsp90-FAM-6, F1-Hsp90-HEX-5, M2- Hsp90-Cyan-5) and 1.25 U of AmpliTaq Gold. The reaction conditions were the same as those used for the standard curves.

All molecular beacon RT-PCR multiplex samples were run in duplicate, or triplicate and the assays were repeated at least once with similar results.

## Results

### Design and optimization of PCR primers and molecular beacon probes

**Figure 1:**
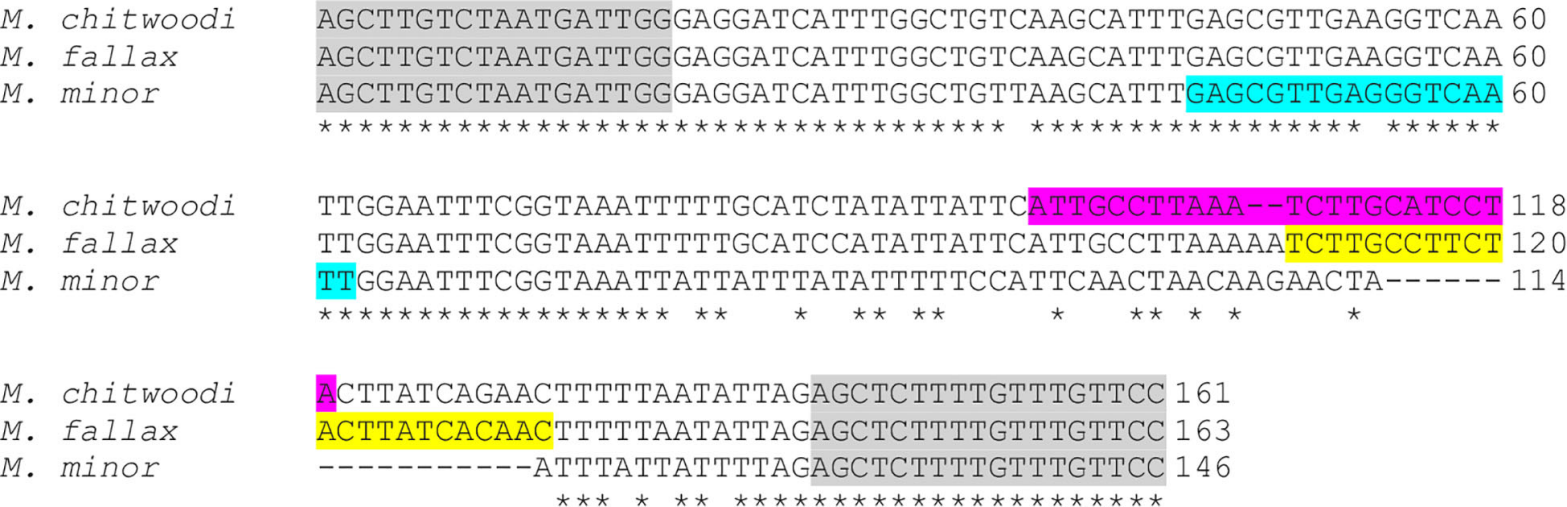
Alignment of partial *HSP90* gene sequences for *M. chitwoodi*, *M. fallax*, and *M. minor*. Universal primer sequences are highlighted in gray. Variable regions targeted by the beacons are highlighted in teal for *M. minor*, magenta for *M. chitwoodi*, and yellow for *M. fallax*. Asterisks indicate conserved regions between all three species, gaps indicate missing nucleotides according to the alignment.

The molecular beacon probes were based on the heat shock protein 90 (*HSP90)* sequence information in Genbank for *M. chitwoodi*, *M. fallax*, and *M. minor* (**Table 1**). This gene was chosen because the sequence is polymorphic between the three species, and the gene had been previously used in a PCR-based nematode identification assay (Nischwitz et al., 2013; Skantar and Carta, 2004). Primers were designed that were specific for the conserved *HSP90* sequence in *M. chitwoodi, M. fallax*, and *M. minor*, but span the polymorphic regions between these species within the gene (**Figure 1**). The *HSP90* gene was amplified using the genomic DNA (gDNA) of *M. chitwoodi race 1, M. fallax*, and *M. minor* as the PCR templates. Sequencing the amplicons confirmed that the predicted polymorphic regions were present in the *HSP90* sequence of each species.

**Figure 2:**
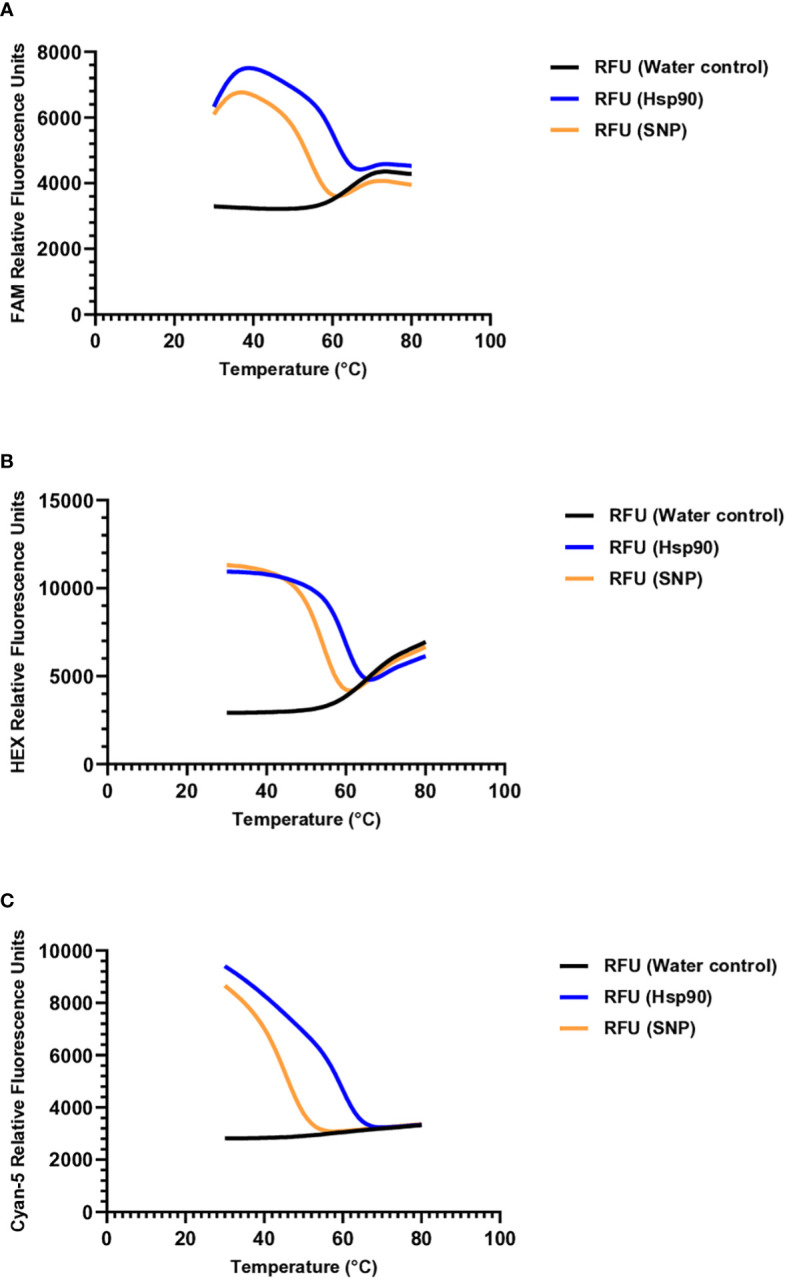
Denaturation curves of molecular beacon probes **(A)** *M. chitwoodi* beacon C1-Hsp90- FAM-6 in the presence of excess complementary template, SNP template, or no template **(B)** *M. fallax* beacon F1-Hsp90-HEX-5 in the presence of excess complementary template, SNP template, or no template. **(C)** *M. minor* beacon M2-Hsp90-Cyan-5 in the presence of excess complementary template, SNP template, or no template.

Molecular beacons were designed to target the polymorphic regions in each species, with the beacons hybridizing to the complementary sequences shown in **Table 2**. To determine the optimal annealing temperature for each molecular beacon probe, a melting curve analysis was performed using oligonucleotides complementary to the molecular beacon probe sequence and oligonucleotides with a single nucleotide substitution (**Table 2**, **Figure 2**). The optimal annealing temperature for *M. chitwoodi*, *M. fallax*, and *M. minor* molecular beacon probes was 54°C.

**Figure 3:**
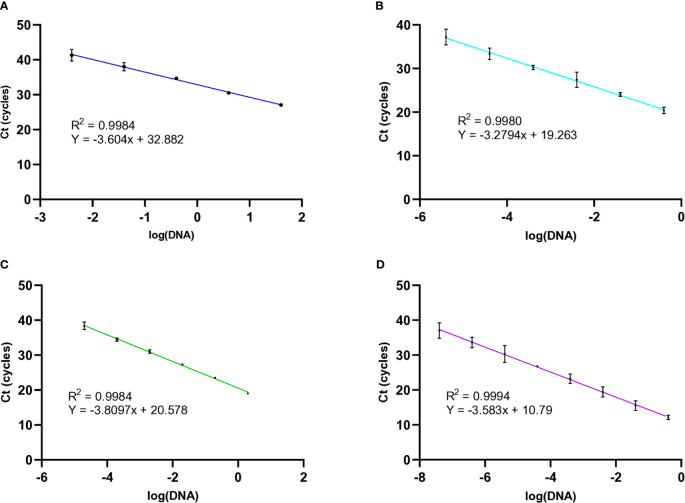
Standard curves of known concentrations of templates from *M. chitwoodi, M, fallax*, and *M. minor* with 95% confidence intervals **(A)** The relationship between Ct values and the natural log of *M. chitwoodi* race 1 gDNA from 40 ng to 4 pg (n = 3). **(B)** *M. chitwoodi HSP90* plasmid DNA template from 2 ng to 20 fg (n = 3). **(C)** *M. fallax HSP90* plasmid DNA template from 0.2 ng to 20 fg (n = 2). **(D)** *M. minor HSP90* plasmid DNA template from 0.4 ng to 0.04 fg (n = 3).

To measure the amplification efficiency of the molecular beacon RT-PCRs, the cycle threshold (Ct) values were obtained over a range of template concentrations for all three nematode species. This information was used to generate standard curves at an annealing temperature of 54°C. First, *M. chitwoodi* race 1 genomic DNA was used as the PCR template with DNA concentrations ranging from 40 ng to 4 pg. When using *M. chitwoodi* race 1 DNA template, the molecular beacon RT-PCR assay had an efficiency of 89% and an R^2^ = 0.9984 (**Figure 3A**). The commonly occurring *M. chitwoodi* Race 1 (Brown et al., 2009) was used as the template for subsequent PCRs and molecular beacon RT-PCR assays (**Figure 3A**). DNA from Race 2 and the pathotype Race 1 Roza of *M. chitwoodi* generated similar standard curves (**Figure S1**), indicating that the molecular beacon RT-PCR assay could detect *M. chitwoodi*, including isolates of the species common in Washington (Bali et al., 2021a).

Next, a fragment of the *M. chitwoodi HSP90* gene was amplified using the F-HSP90 and R-HSP90 primers. This amplicon was cloned to generate the “*M. chitwoodi HSP90* plasmid,” which was used as the PCR template for further reactions. The efficiency of the molecular beacon RT-PCR using the *M. chitwoodi HSP90* plasmid as the PCR template was compared to using genomic DNA as the reaction template. By calculating the slope of the standard curve, the PCR efficiency for *M. chitwoodi HSP90* plasmid was 101% with an R^2^ = 0.9980 (**Figure 3B**). This was better, but comparable to the reaction efficiencies using *M. chitwoodi* genomic DNA as the reaction template (efficiency of 89% and an R^2^ = 0.9984).

Cloned *HSP90* fragments from *M. fallax* and *M. minor* were also used as template for the molecular beacon RT-PCR assays. The *M. minor* molecular beacon RT-PCR had an efficiency of 90% and an R2 = 0.9994 (**Figure 3D**), and the *M. fallax* molecular beacon RT-PCR had an efficiency of 83% with an R^2^ = 0.9984 (**Figure 3C**).

### The molecular beacon probes are specific for the target nematode

The *M. chitwoodi* molecular beacon probe could detect the *M. chitwoodi* amplicon, but not the amplicons from *M. fallax* and *M. minor* in a molecular beacon RT-PCR (**Figure S2A**). The same was true for the other molecular beacon probes; the *M. fallax* probe could only hybridize to the *M. fallax* amplicon (**Figure S2B**) and the *M. minor* probe could only hybridize to the *M. minor* amplicon (**Figure S2C**).

*Meloidogyne hapla, M. incognita*, *M. javanica*, and *M. arenaria* are the four major species of root- knot nematodes found worldwide. When the *HSP90* sequences of *M. hapla*, *M. incognita*, *M. javanica*, or *M. arenaria* were aligned with the *HSP90* sequences from *M. chitwoodi, M. fallax* and *M. minor*, the probes were specific to their respective nematode (**Figure S3**). When qPCR was run using gDNA from *M. hapla*, *M. incognita*, *M. javanica*, or *M. arenaria* none of the molecular beacon probes produced fluorescence (**Figure S4**), indicating that there is no off-target binding to these other root-knot nematode species.

### *HSP90* molecular beacon probes detect the presence of a single juvenile *of M. chitwoodi*, *M. fallax*, or *M. minor*

**Figure 4:**
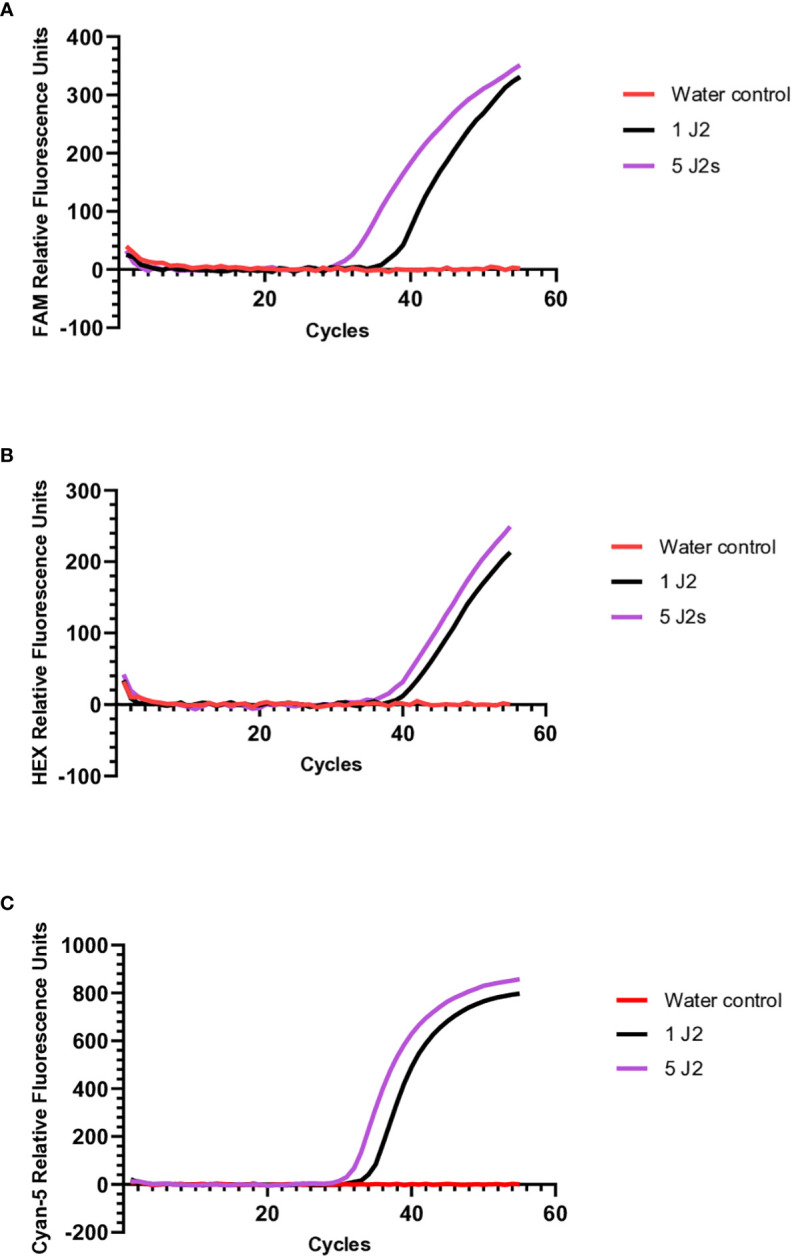
Molecular beacon RT-PCR amplification curves using crude extracts from 1 or 5 J2s of **(A)** *M. chitwoodi* **(B)** *M. fallax*, or **(C)** *M. minor* as the reaction template.

To validate whether the molecular beacon RT-PCR assays could detect *M. chitwoodi*, *M. fallax*, or *M. minor* using DNA isolated directly from juveniles, crude extracts from 1 or 5 J2s of each species was used as the template for molecular beacon RT-PCR. The *M. minor* assay was the most sensitive with an average Ct of 35.7 (standard error of means (SEM) = 0.22, n = 7) for a single J2, and an average Ct of 32.7 for 5 J2s (SEM = 0.46, n = 2) (**Figure 4C**). The *M. fallax* assay was the least sensitive, with a single J2 sample having an average Ct of 43.6 (SEM = 1.16, n = 8) and 5 J2s having an average Ct of 39.8 (SEM = 1.04, n = 2) (**Figure 4B**). Finally, the molecular beacon RT- PCR for *M. chitwoodi* produced an average Ct for a single J2 of 38.9 (SEM = 0.64, n = 10) and a Ct of 34.7 for 5 J2s (SEM = 0.83, n = 2) (**Figure 4A**). All J2s digests were positive for all three species satisfying the 95% positive replicate criterion suggested by Forootan et al. (2017) for qPCR limit of detection. In the negative controls, no significant fluorescence was observed. These results demonstrate the sensitivity of the molecular beacon probes and their ability to detect the presence of *M. chitwoodi*, *M. fallax*, or *M. minor* from the crude extract of a single juvenile.

### Multiplex PCR with the molecular beacon probes

**Figure 5:**
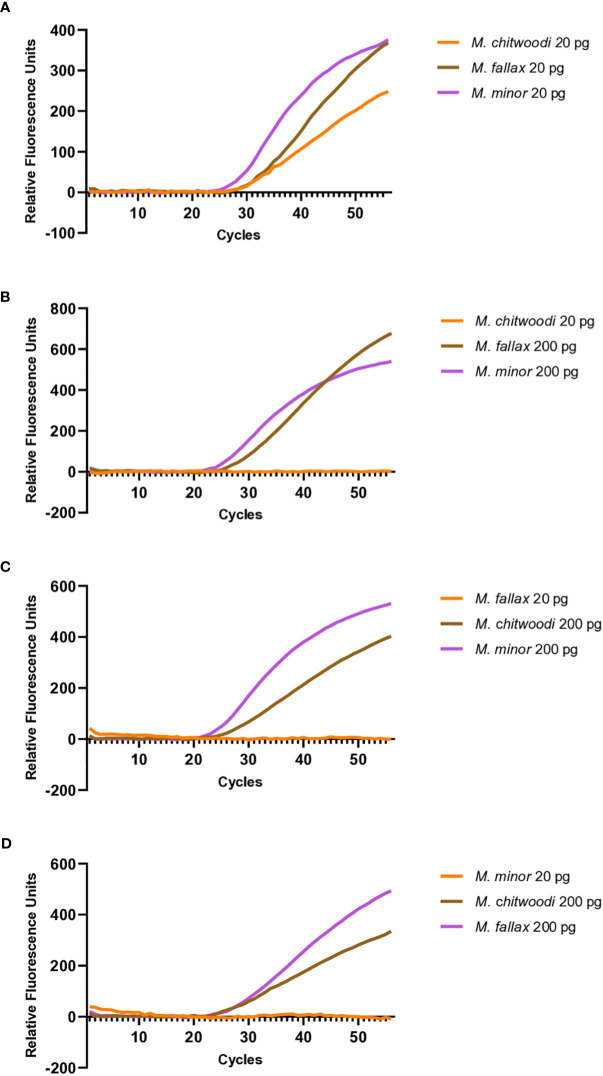
Multiplexed molecular beacon RT-PCR assays containing equal or unequal parts *M. chitwoodi*, *M. fallax*, and *M. minor* plasmid template. **(A)** Amplification curves from 20 pg of *M. chitwoodi* (average Ct = 31.3, SEM = 0.11, n = 6), *M. fallax* (average Ct = 30.9, SEM = 0.20, n = 6), and *M. minor* (average Ct = 30.1, SEM = 0.09, n = 6) in a single reaction **(B)** Amplification curves from 20 pg of *M. chitwoodi* (no amplification, n = 6), 200 pg *M. fallax* (average Ct = 26.8, SEM = 0.62, n = 6), and 200 pg *M. minor* (average Ct = 26.3, SEM = 0.39, n = 6) in a single reaction **(C)** Amplification curves from 20 pg of *M. fallax* (no amplification, n = 6), 200 pg *M. chitwoodi* (average Ct = 27.0, SEM = 0.50, n = 6), and 200 pg *M. minor* (average Ct = 25.1, SEM = 0.13, n = 6) in a single reaction. **(D)** Amplification curves from 20 pg of *M. minor* (no amplification, n = 6), 200 pg *M. chitwoodi* (average Ct = 26.6, SEM = 0.24, n = 6), and 200 pg *M. fallax* (average Ct = 26.6, SEM = 0.20, n = 6) in a single reaction.

**Figure 6:**
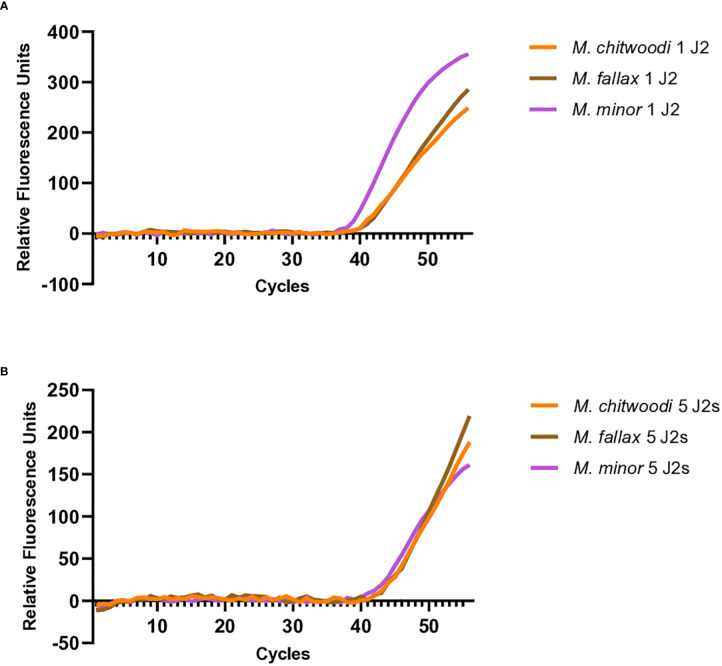
Multiplexed molecular beacon RT-PCR assays containing an equal mix of 1 or 5 J2s from *M. chitwoodi*, *M. fallax*, and *M. minor* as template. **(A)** Amplification curves from 1 J2 of *M. chitwoodi* (average Ct = 42.3, SEM = 0.43, n = 10), *M. fallax* (average Ct = 43.7, SEM = 0.78, n = 10), and *M. minor* (average Ct = 41.1, SEM = 0.69, n = 10) in a single reaction. **(B)** Amplification curves from 5 J2s of *M. chitwoodi* (average Ct = 42.4, SEM = 0.63, n = 4), *M. fallax* (average Ct = 42.7, SEM = 0.92, n = 4), and *M. minor* (average Ct = 42.7, SEM = 0.62, n = 4) in a single reaction.

Further investigation was carried out to see if the molecular beacon RT-PCR assay could detect the nematode of interest when there is a mixture of nematodes in the reaction. When 20 pg of *M. chitwoodi*, *M. fallax*, and *M. minor HSP90* templates were mixed in a single reaction containing all three beacon probes, it was possible to detect each species (**Figure 5A**). Detection of all three species was also possible using crude extracts from digested J2s when all 3 species were mixed, either as single J2s or as 5 J2s each (**Figure 6**).

To investigate how the assay performed when templates were not present in equal parts, mixes of *M. chitwoodi*, *M. fallax*, and *M. minor HSP90* plasmid templates were made that had one species present at 20 pg, and the other two species present at 200 pg. The templates present at 200 pg were reliably detected, but the template at 20 pg could not be detected in these reactions (**Figures 5B–D**).

Overall, the data suggests that the molecular beacon RT-PCR assay can detect each species in a multiplexed reaction. However, an important caveat is that the sample detection is inhibited for the target species if the other two species are in relative excess in concentration.

## Discussion

Nematode identification is a critical component for biosecurity, particularly when dealing with regulated nematodes. *Meloidogyne minor, M. chitwoodi* and *M. fallax* juveniles can be difficult to distinguish from each other, making morphological-based identification challenging. We designed a molecular beacon RT-PCR assay to detect *M. chitwoodi, M. minor*, and *M. fallax*. The assay is species-specific, easy to perform, rapid, and reliable. It is also able to detect small amounts of DNA and could identify the species using a single J2 as the reaction template. This means it can be adapted for identifying J2s extracted from soil or from J2s hatched from eggs (Adam et al., 2007). This assay can be very useful because individual J2s can be picked and then used directly to identify if it is one of three species. If all three beacons are used on a single J2, a positive or negative identification of either *M. chitwoodi*, *M. fallax*, or *M. minor* can be obtained in a reaction time under 2 hours. This assay will help to determine the distribution of these nematodes and prevent their spread to new potato growing regions.

A previous comparison between TaqMan and molecular beacon probes for analyzing single nucleotide polymorphisms (SNPs) in human DNA found that molecular beacon probes are better in detecting sequence variants; in a panel of DNA samples, the molecular beacon probes were more reliable in detecting GC-rich targets compared to the TaqMan probes designed for that region (Täpp et al., 2000). In addition, the data showed that the molecular beacon probes could better detect minority sequence variants over a wider range of template concentrations compared to the TaqMan assay, suggesting that the molecular beacon probes offer advantages in sensitivity and robustness over TaqMan assays (Täpp et al., 2000). Our results indicate that the molecular beacon probes we developed for *M. chitwoodi* and *M. fallax* are more sensitive than the previously developed TaqMan assay for these two species (Zijlstra and Van Hoof, 2006). Our molecular beacon RT-PCR assays could detect DNA concentrations as low as 0.04 fg for *M. minor* and 4 fg and 2 fg for *M. chitwoodi* and *M. fallax*, respectively, which is better than or comparable to previous TaqMan molecular beacon RT-PCR assays (Braun-Kiewnick and Kiewnick, 2018; Zijlstra and Van Hoof, 2006; Weerdt et al., 2011). The *M. chitwoodi* and *M. minor* assays both had PCR efficiencies of ≥ 90%. This is an improvement in efficiency for *M. minor* (Weerdt et al., 2011) and a similar efficiency for *M. chitwoodi* compared to the previously published TaqMan assays (Zijlstra and Van Hoof, 2006). The *M. fallax* assay had the lowest PCR efficiency of the three at 83%. However, 83% is still a relatively good efficiency compared to previously published PCR assays for root-knot nematodes. For example, the TaqMan assay for *M. minor* had an efficiency of 62% (Weerdt et al., 2011). The performance of the molecular beacon RT-PCR assays indicate that the assay has a high sensitivity, specificity (the probes only detect the target species) and reproducibility over multiple experiments. While this assay is species-specific, standard curves using Race 1, Race 2, or Race 1 Roza gDNA from *M. chitwoodi* showed that this assay cannot distinguish between the *M. chitwoodi* isolates. Therefore, this the assay would be suitable for general *M. chitwoodi* detection despite the fact that the known races in Washington differ genetically (Bali et al., 2021b).

Multiplexing would allow for the simultaneous detection of the three species in one tube. The multiplexing experiments showed that we could detect each species of nematode when similar quantities of template of the three species were present. Because the PCR efficiencies were high and because the PCR primers are targeting the same sequences in all three species, when all three species are present in similar amounts, the amount of target amplicons produced from each species should be similar, resulting in detectable amplicons. However, we found that our ability to detect a species was compromised if its DNA template was present at relatively low ratios (≥1:10) compared to the other species. Although the sensitivity of multiplexing is limited by the relative quantities of DNA template from each of the three species, it is still a useful tool for the sensitive detection and identification of *M. chitwoodi*, *M. fallax*, and *M. minor*.

This assay provides a simple and rapid molecular protocol for distinguishing *M. chitwoodi*, *M. fallax*, and *M. minor* from one another using as little as a single J2 as the reaction template. The assays described here use DNA isolated from juvenile(s) since they are commonly found in soil samples, but DNA could be isolated from eggs and adult nematodes and used as the reaction template. This is the first molecular method that reliably identifies these three species of potato-infecting root-knot nematodes. It could be used by diagnosticians and extension agents as a tool for tracking the spread of *M. chitwoodi*, *M. fallax* and *M. minor* in the USA.

## Data availability statement

The raw data supporting the conclusions of this article will be made available by the authors, without undue reservation.

## Author contributions

SA performed the experiments and statistical analyses. SA and CG designed the experiments and wrote the manuscript text. Both authors contributed to the article and approved the submitted version.

## References

[B1] Adam M. A. M. Phillips M. S. Blok V. C. (2007). Molecular diagnostic key for identification of single juveniles of seven common and economically important species of root-knot nematode (*Meloidogyne* spp.). Plant Pathol. 56, 190–197. doi: 10.1111/j.1365-3059.2006.01455.x

[B2] Bali S. Hu S. Vining K. Brown C. Mojtahedi H. Zhang L.. (2021a). Nematode genome announcement: Draft genome of *Meloidogyne chitwoodi*, an economically important pest of potato in the pacific Northwest. Mol. Plant Microbe Interact. 34, 981–986. doi: 10.1094/MPMI-12-20-0337-A 33779267

[B3] Bali S. Zhang L. Franco J. Gleason C. (2021b). Biotechnological advances with applicability in potatoes for resistance against root-knot nematodes. Curr. Opin. Biotechnol. 70, 226–233. doi: 10.1016/j.copbio.2021.06.010 34217954

[B4] Baum T. J. Gresshoff P. M. Lewis S. A. Dean R. A. (1994). Characterization and phylogenetic analysis of four root-knot nematode species using DNA amplification fingerprinting and automated polyacrylamide gel electrophoresis. Mol. Plant Microbe Interact. 7, 39–47. doi: 10.1094/MPMI-7-0039

[B5] Blok V. C. Phillips M. S. Fargette M. (1997). Comparison of sequences from the ribosomal DNA intergenic region of *Meloidogyne mayaguensis* and other major tropical root-knot nematodes. J. Nematol. 29, 16–22. 19274129 PMC2619761

[B6] Braun-Kiewnick A. Kiewnick S. (2018). Real-time PCR, a great tool for fast identification, sensitive detection and quantification of important plant-parasitic nematodes. Eur. J. Plant Pathol. 152, 271–283. doi: 10.1007/s10658-018-1487-7

[B7] Brown C. R. Mojtahedi H. Zhang L. H. Riga E. (2009). Independent resistant reactions expressed in root and tuber of potato breeding lines with introgressed resistance to *Meloidogyne chitwoodi* . Phytopathology 99, 1085–1089. doi: 10.1094/PHYTO-99-9-1085 19671011

[B8] Castagnone-Sereno P. (2000). Use of satellite DNA for specific diagnosis of the quarantine root- knot nematodes *Meloidogyne chitwoodi* and *M. fallax* . Bull. OEPP 30, 581–584. doi: 10.1111/j.1365-2338.2000.tb00951.x 18944750

[B9] Castagnone-Sereno P. Leroy F. Bongiovanni M. Zijlstra C. Abad P. (1999). Specific diagnosis of two root-knot nematodes, *Meloidogyne chitwoodi* and *M. fallax*, with satellite DNA probes. Phytopathology 89, 380–384. doi: 10.1094/PHYTO.1999.89.5.380 18944750

[B10] European and Mediterranean Plant Protection Organization [EPPO] (2022) *Meloidogyne fallax.* EPPO datasheets on pests recommended for regulation. Available at: https://gd.eppo.int (Accessed December 23, 2022).

[B11] FAO. (2017). Crops and livestock products. Available at: https://www.fao.org/faostat/en/#data/QCL (Accessed 04-05-2021).

[B12] Forootan A. Sjöback R. Björkman J. Sjögreen B. Linz L. Kubista M. (2017). Methods to determine limit of detection and limit of quantification in quantitative real-time PCR (qPCR). Biomol. Detect. Quantif. 12, 1–6. doi: 10.1016/j.bdq.2017.04.001 28702366 PMC5496743

[B13] Gamel S. Huchet E. Le Roux-Nio A.-C. Anthoine G. (2014). Assessment of PCR-based tools for the specific identification of some temperate *Meloidogyne* species including *M. chitwoodi*, *M. fallax* and *M. minor* . Eur. J. Plant Pathol. 138, 807–817. doi: 10.1007/s10658-013-0355-8

[B14] Giorgi C. D. Sialer M. F. Vito M. D. Lamberti F. (1994). Identification of plant-parasitic nematodes by PCR amplification of DNA fragments. Bull. OEPP 24, 447–451. doi: 10.1111/j.1365-2338.1994.tb01403.x

[B15] Gross S. M. Williamson V. M. (2011). Tm1: A mutator/foldback transposable element family in root-knot nematodes. PloS One 6, 9. doi: 10.1371/journal.pone.0024534 PMC3169594 21931741

[B16] Hadziavdic K. Lekang K. Lanzen A. Jonassen I. Thompson E. M. Troedsson E. (2014). Characterization of the 18S rRNA gene for designing universal eukaryote specific primers. PLoS One 9, 2. doi: 10.1371/journal.pone.0087624 PMC3917833 24516555

[B17] Han H. Cho M.-R. Jeon H.-Y. Lim C.-K. Jang H.-I. (2004). PCR-RFLP identification of three major *Meloidogyne* species in Korea. J. Asia Pac. Entomol. 7, 171–175. doi: 10.1016/S1226-8615(08)60212-5

[B18] Harris T. S. Sandall L. J. Powers T. O. (1990). Identification of single *Meloidogyne* juveniles by polymerase chain reaction amplification of mitochondrial DNA. J. Nematol. 22, 518–524. 19287752 PMC2619072

[B19] Ingham R. E. Hamm P. B. Baune M. David N. L. Wade N. M. (2007). Control of *Meloidogyne chitwoodi* in potato with shank-injected metam sodium and other nematicides. J. Nematol. 39, 161–168. 19259485 PMC2586488

[B20] Ingham R. E. Hamm P. B. Williams R. E. Swanson W. H. (2000). Control of *Meloidogyne chitwoodi* in potato with fumigant and nonfumigant nematicides. J. Nematol. 32, 556–565. 19271010 PMC2620491

[B21] Kantor M. Handoo Z. Kantor C. Carta L. (2022). Top ten most important U.S.-regulated and emerging plant-parasitic nematodes. Horticulturae 8, 208. doi: 10.3390/horticulturae8030208

[B22] Karssen G. (1996). Description of *Meloidogyne fallax* n. sp. (Nematoda: Heteroderidae), a root- knot nematode from the Netherlands. Fundam. Appl. Nematol. 19, 593–599.

[B23] Karssen G. Bolk R. J. van Aelst A. C. van den Beld I. Kox L. F. F. Korthals G. W.. (2004). Description of *Meloidogyne minor* n. sp. (Nematoda: Meloidogynidae), a root-knot nematode associated with yellow patch disease in golf courses. Nematology 6, 59–72. doi: 10.1163/156854104323072937

[B24] Koenning S. R. Overstreet C. Noling J. W. Donald P. A. Becker J. O. Fortnum B. A. (1999). Survey of crop losses in response to phytoparasitic nematodes in the united states for 1994. J. Nematol. 31, 587–618. 19270925 PMC2620402

[B25] Lima F. S. O. Mattos V. S. Silva E. S. Carvalho M. A. S. Teixeira R. A. Silva J. C.. (2018). “Nematodes affecting potato and sustainable practices for their management,” in Potato - from incas to all over the world. Ed. Yildiz M. (London: IntechOpen). doi: 10.5772/intechopen.73056

[B26] Mojtahedi H. Brown C. R. Riga E. Zhang L. H. (2007). A new pathotype of *Meloidogyne chitwoodi* race 1 from Washington state. Plant Dis. 91, 1051. 10.1094/PDIS-91-8-1051A 30780441

[B27] McClure M. A. Nischwitz C. Skantar A. M. Schmitt M. E. Subbotin S. A. (2012). Root- knot nematodes in golf course greens of the Western united states. Plant Dis. 96, 635–647. doi: 10.1094/PDIS-09-11-0808 30727525

[B28] Morris K. Horgan F. Downes M. Griffin C. (2011). The effect of temperature on hatch and activity of second-stage juveniles of the root-knot nematode, *Meloidogyne minor*, an emerging pest in north-west Europe. Nematology 13, 985–993. doi: 10.1163/138855411X571902

[B29] Morris K. Horgan F. G. Griffin C. T. (2013). Spatial and temporal dynamics of *Meloidogyne minor* on creeping bentgrass in golf greens. Plant Pathol. 62, 1166–1172. doi: 10.1111/ppa.12025

[B30] Nischwitz C. Skantar A. Handoo Z. A. Hult M. N. Schmitt M. E. McClure M. A. (2013). Occurrence of *Meloidogyne fallax* in north America, and molecular characterization of *M. fallax* and *M. minor* from U.S. golf course greens. Plant Dis. 97, 1424–1430. doi: 10.1094/PDIS-03-13-0263-RE 30708461

[B31] Orui Y. (1998). Identification of Japanese species of the genus *Meloidogyne* (Nematoda: Meloidogynidae) by PCR-RFLP analysis. Appl. Entomol. Zool. 33, 43–51. doi: 10.1303/aez.33.43

[B32] Petersen D. J. Zijlstra C. Wishart J. (1997). Specific probes efficiently distinguish root-knot nematode species using signature sequences in the ribosomal intergenic spacer. Fundam. Appl. Nematol. 20, 619–626.

[B33] Pinkerton J. N. Santo G. S. Mojtahedi H. (1991). Population dynamics of *Meloidogyne chitwoodi* on russet Burbank potatoes in relation to degree-day accumulation. J. Nematol. 23, 283–290. 19283128 PMC2619162

[B34] Powers T. O. Harris T. S. (1993). A polymerase chain reaction method for identification of five major *Meloidogyne* species. J. Nematol. 25, 1–6. 19279734 PMC2619349

[B35] Powers T. O. Todd T. C. Burnell A. M. Murray P. C. B. Fleming C. C. Szalanski A. L.. (1997). The rDNA internal transcribed spacer region as a taxonomic marker for nematodes. J. Nematol. 29, 441–450. 19274180 PMC2619808

[B36] Qiu J. J. Westerdahl B. B. Anderson C. Williamson V. M. (2006). Sensitive PCR detection of *Meloidogyne arenaria*, *M. incognita*, and *M. javanica* extracted from soil. J. Nematol. 38, 434–441. 19259460 PMC2586468

[B37] Sasser J. N. Freckman D. W. (1987). “A world perspective on nematology: The role of the society,” in Vistas on nematology. Eds. Veech J. A. Dickson D. W. (Hyattsville, MD: Society of Nematologists, Inc) 7–20.

[B38] Skantar A. M. Carta L. K. (2004). Molecular characterization and phylogenetic evaluation of the hsp90 gene from selected nematodes. J. Nematol. 36, 466–480. 19262827 PMC2620790

[B39] Suffert M. Giltrap N. (2012). EPPO workshop on *Meloidogyne chitwoodi* and meloidogyne fallax, (2011-03-08): Importance for potato production and experience of management in EPPO countries. Bull. OEPP 42, 117–121. doi: 10.1111/j.1365-2338.2012.02528.x

[B40] Täpp I. Malmberg L. Rennel E. Wik M. Syvänen A. C. (2000). Homogeneous scoring of single-nucleotide polymorphisms: Comparison of the 5’-nuclease TaqMan assay and molecular beacon probes. BioTechniques 28, 732–738. doi: 10.2144/00284rr02 10769752

[B41] Thoden T. C. Korthals G. W. Visser J. van Gastel-Topper W. (2012). A field study on the host status of different crops for *Meloidogyne minor* and its damage potential on potatoes. Nematology 14, 277–284. doi: 10.1163/156854111X594965

[B42] Van Der Beek J. G. Vereijken P. F. G. Poleij L. M. Silfhout C. H. V. (1998). Isolate-by- cultivar interaction in root-knot nematodes *Meloidogyne hapla*, *M. chitwoodi*, and *M. fallax* on potato. Can. J. Bot. 76, 75–82. doi: 10.1139/b97-161

[B43] Van Megen H. van den Elsen S. Holterman M. Karssen G. Mooyman P. Bongers T.. (2009). A phylogenetic tree of nematodes based on about 1200 full-length small subunit ribosomal DNA sequences. Nematology 11, 927–950. doi: 10.1163/156854109X456862

[B44] Van Meggelen J. C. Karssen G. Janssen G. J. W. Verkerk-Bakker B. Janssen R. (1994). New race of *Meloidogyne chitwoodi* golden, O’Bannon, Santo & Finley 1980? Fundam. Appl. Nematol. 17, 93–96.

[B45] Vandenbossche B. Viaene N. de Sutter N. Maes M. Karssen G. Bert W. (2011). Diversity and incidence of plant-parasitic nematodes in Belgian turf grass. Nematology 13, 245–256. doi: 10.1163/138855410X517084

[B46] Viaene N. (2014). Nematodes: An increasing threat for the potato crop in Europe? Potato Res. 57, 335–338. doi: 10.1007/s11540-015-9284-6

[B47] Vrain T. C. Petersen D. J. (1996). Rapid identification of *Meloidogyne chitwoodi*, *M. hapla*, and *M. fallax* using PCR primers to amplify their ribosomal intergenic spacer. Fundam. Appl. Nematol. 19, 601–605.

[B48] Vrain T. C. Wakarchuk D. A. Levesque A. C. Hamilton R. L. (1992). Intraspecific rDNA restriction fragment length polymorphism in the *Xiphinema americanum* group. Fundam. Appl. Nematol. 15, 563–573.

[B49] Waeyenberge L. Ryss A. Moens M. Pinochet J. Vrain T. (2000). Molecular characterisation of 18 *Pratylenchus* species using rDNA restriction fragment length polymorphism. Nematology 2, 135–142. doi: 10.1163/156854100509024

[B50] Weerdt M. D. Kox L. Waeyenberge L. Viaene N. Zijlstra C. (2011). A real-time PCR assay to identify *Meloidogyne minor* . J. Phytopathol. 159, 80–84. doi: 10.1111/j.1439-0434.2010.01717.x

[B51] Wesemael W. M. L. Taning L. M. Viaene N. Moens M. (2014). Life cycle and damage of the root-knot nematode *Meloidogyne minor* on potato, solanum tuberosum. Nematology 16, 185–192. doi: 10.1163/15685411-00002756

[B52] Wesemael W. Viaene N. Moens M. (2011). Root-knot nematodes (*Meloidogyne* spp.) in Europe. Nematology 13, 3–16. doi: 10.1163/138855410X526831

[B53] Wishart J. Phillips M. S. Blok V. C. (2002). Ribosomal intergenic spacer: a polymerase chain reaction diagnostic for *Meloidogyne chitwoodi*, *M. fallax*, and *M. hapla* . Phytopathology 92, 884–892. doi: 10.1094/PHYTO.2002.92.8.884 18942968

[B54] Williamson V. M. Caswell-Chen E. P. Westerdahl B. B. Wu F. F. Caryl G. (1997). A PCR assay to identify and distinguish single juveniles of *Meloidogyne hapla* and *M. chitwoodi* . J. Nematol. 29, 9–15. 19274128 PMC2619763

[B55] Zasada I. A. Dandurand L.-M. Gleason C. Hagerty C. H. Ingham R. E. (2018). “Plant parasitic nematodes of the pacific Northwest: Idaho, Oregon and Washington,” in Plant parasitic nematodes in sustainable agriculture of north America. Eds. Subbotin S. A. Chitambar J. J. (Cham, Switzerland: Springer International Publishing), 211–239. doi: 10.1007/978-3-319-99585-4_8

[B56] Zeng Y. Ye W. Kerns J. Tredway L. Martin S. Martin M. (2015). Molecular characterization and phylogenetic relationships of plant-parasitic nematodes associated with turfgrasses in north Carolina and south Carolina, united states. Plant Dis. 99, 982–993. doi: 10.1094/PDIS-10-14-1060-RE 30690976

[B57] Zhang L. Gleason C. (2019). Loop-mediated isothermal amplification for the diagnostic detection of *Meloidogyne chitwoodi* and *M. fallax* . Plant Dis. 103, 12–18. doi: 10.1094/PDIS-01-18-0093-RE 30358508

[B58] Zijlstra C. (1997). A fast PCR assay to identify *Meloidogyne hapla*, *M. chitwoodi*, and *M. fallax*, and to sensitively differentiate them from each other and from *M. incognita* in mixtures. Fundam. Appl. Nematol. 20, 505–511.

[B59] Zijlstra C. (2000). Identification of *Meloidogyne chitwoodi*, *M. fallax* and *M. hapla* based on SCAR-PCR: A powerful way of enabling reliable identification of populations or individuals that share common traits. Eur. J. Plant Pathol. 106, 283–290. doi: 10.1023/A:1008765303364

[B60] Zijlstra C. Van Hoof R. A. (2006). A multiplex real-time polymerase chain reaction (TaqMan) assay for the simultaneous detection of *Meloidogyne chitwoodi* and *M. fallax* . Phytopathology 96, 1255–1262. doi: 10.1094/PHYTO-96-1255 18943963

[B61] Zijlstra C. Lever A. E. M. Uenk B. J. van Silfhout C. H. (1995). Differences between ITS regions of isolates of the root-knot nematodes *Meloidogyne hapla* and *M. chitwoodi* . Phytopathology 85, 1231–1237. doi: 10.1094/Phyto-85-1231

