# Supplementary figures and images for: A molecular beacon real-time polymerase chain reaction assay for the identification of M. chitwoodi, M. fallax, and M. minor
Source: Front Plant Sci. 2023 Feb 22;14:1096239. doi: 10.3389/fpls.2023.1096239 (PMC9994647; doi:10.3389/fpls.2023.1096239)

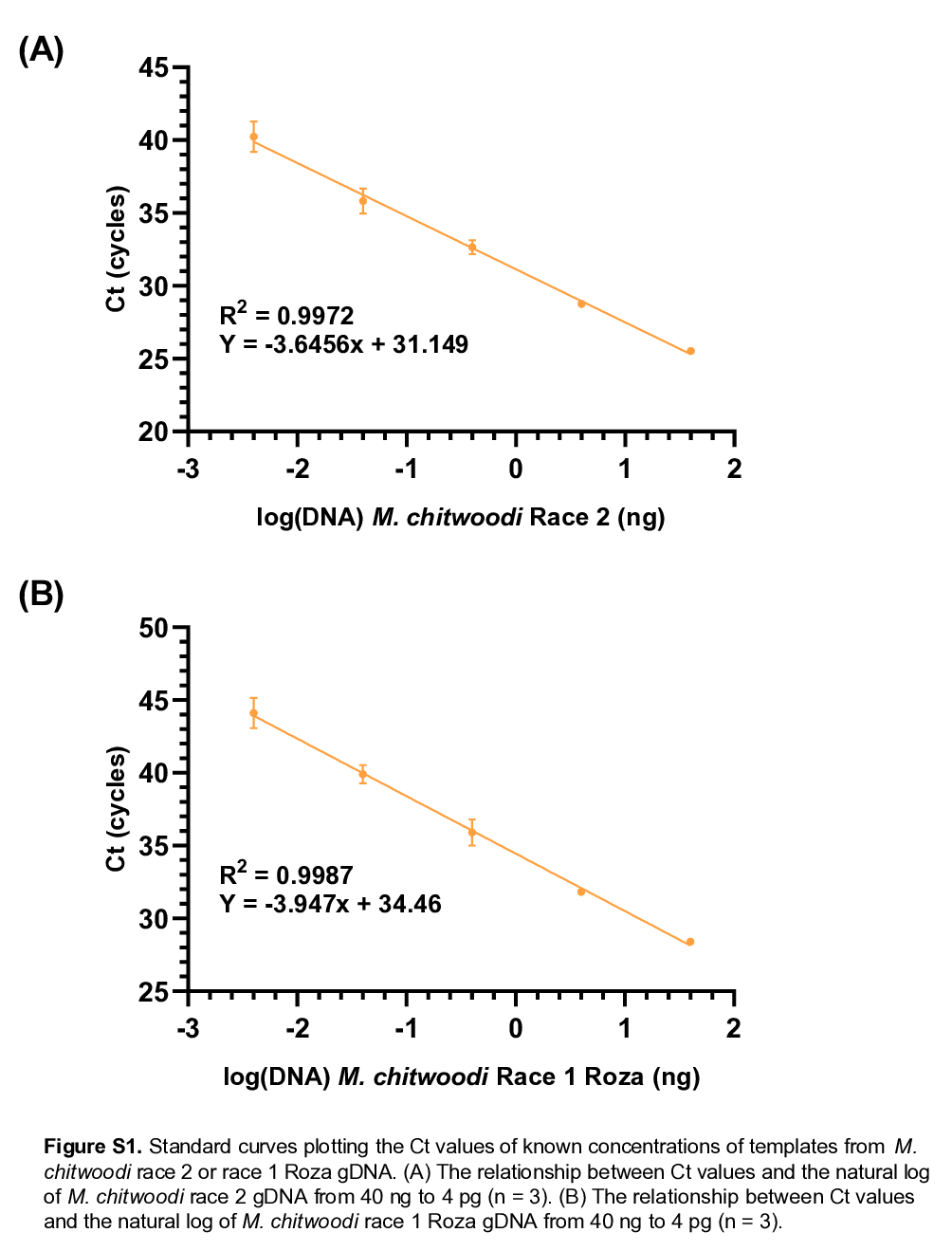

Supplement: Supplementary file 1 [file Image_1.tiff]

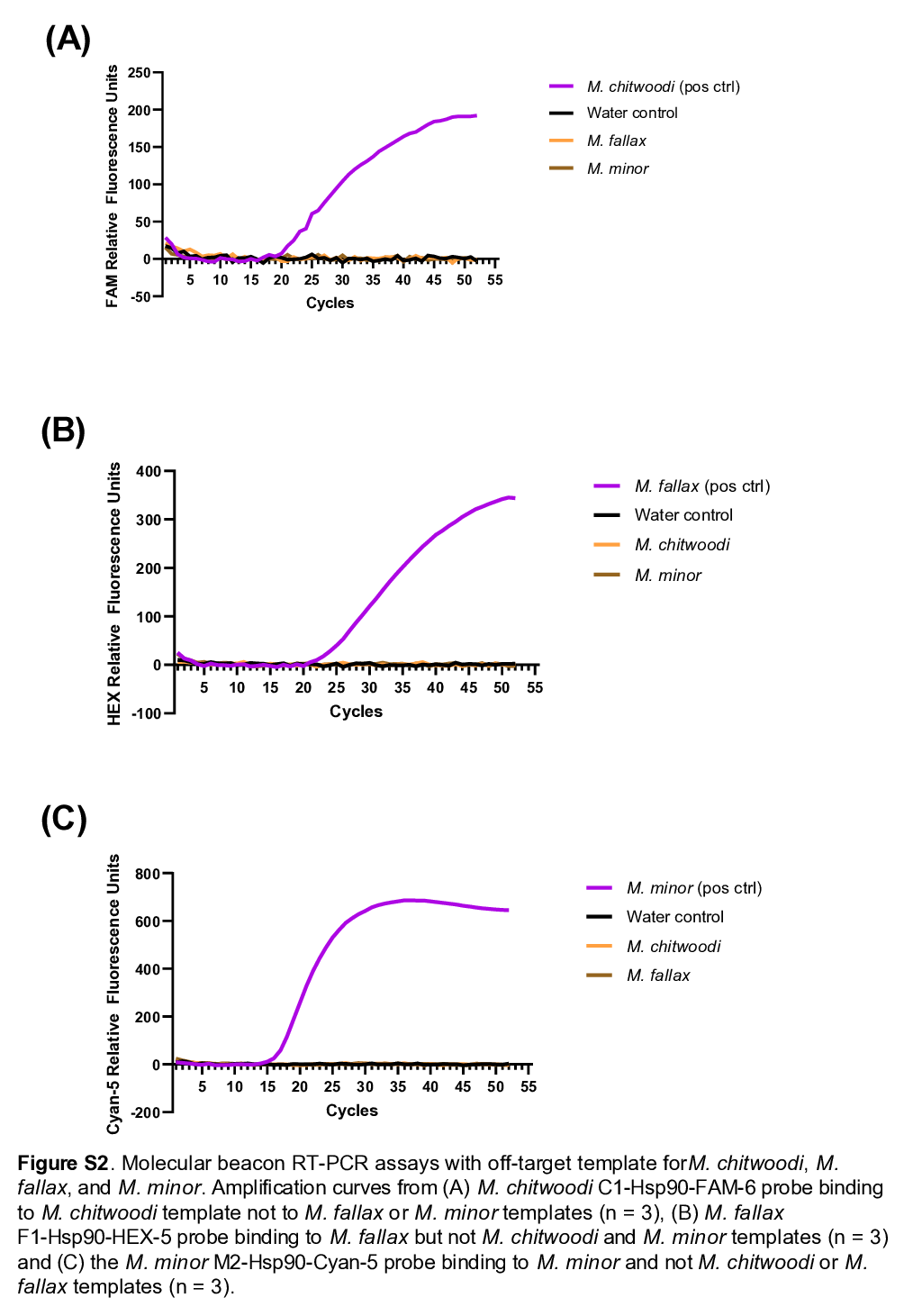

Supplement: Supplementary file 2 [file Image_2.tiff]

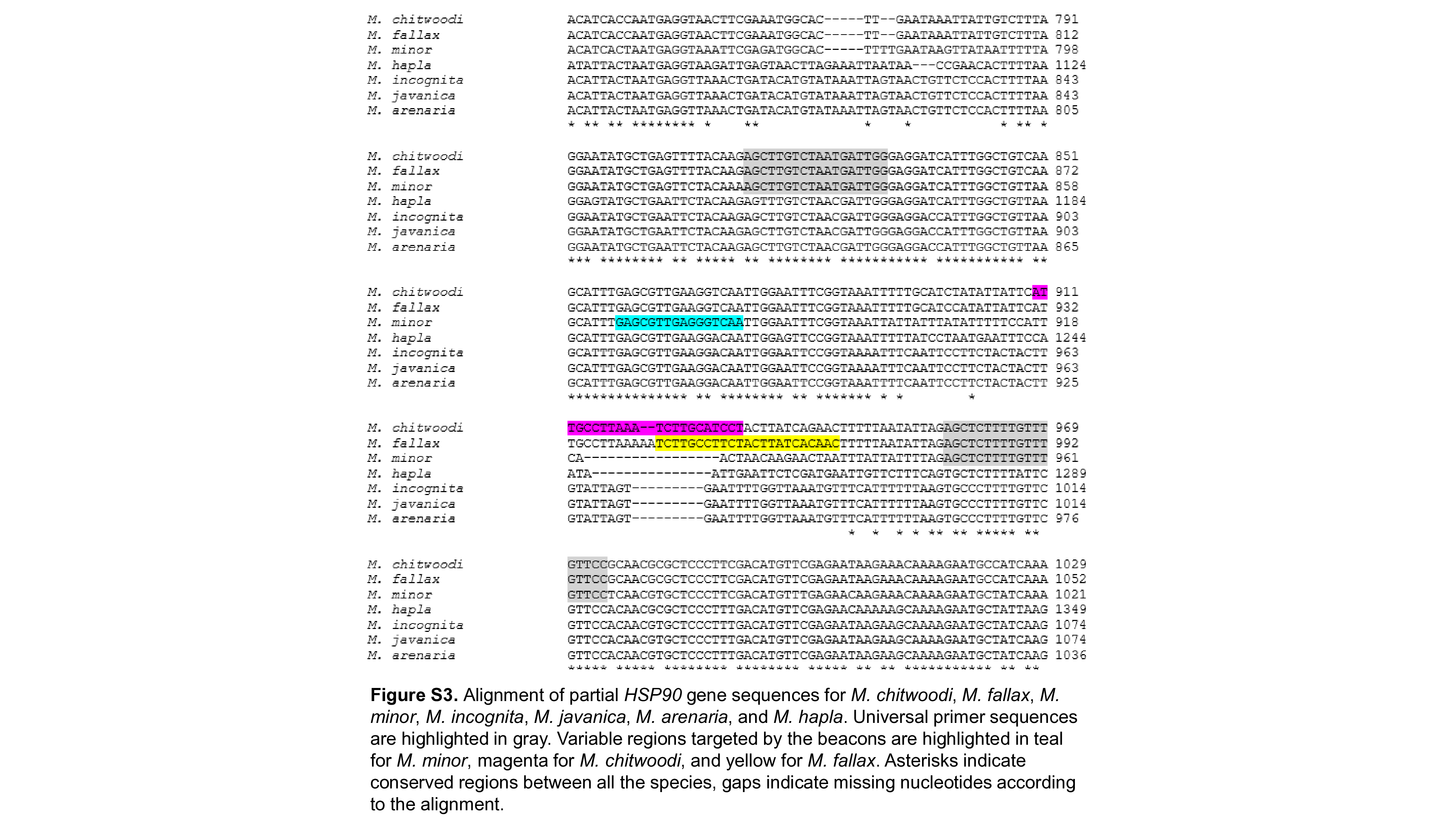

Supplement: Supplementary file 3 [file Image_3.tiff]

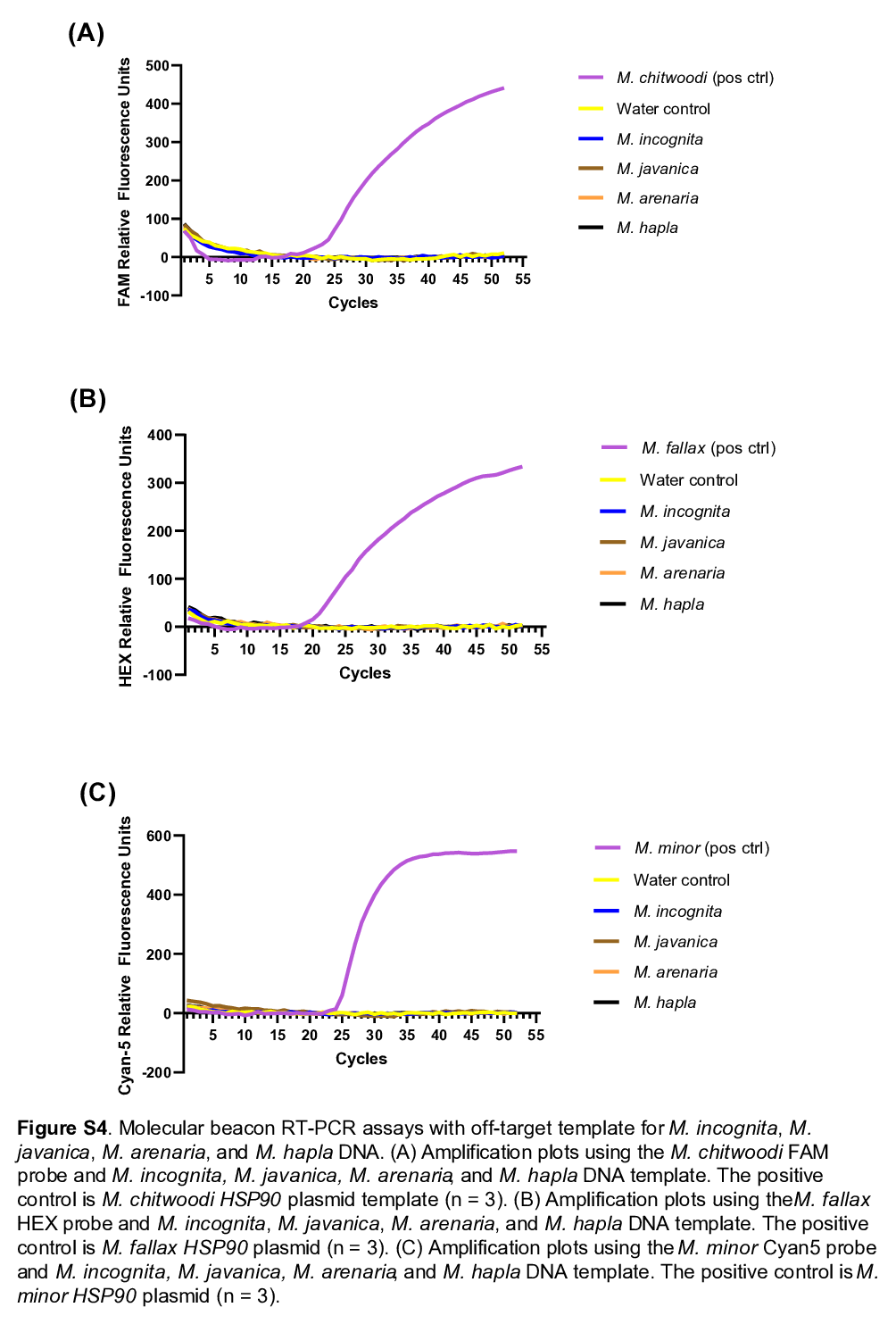

Supplement: Supplementary file 4 [file Image_4.tiff]
